# Evaluation of the thickness of acetabular cartilage by ultrasound in developmental dysplasia of the hip

**DOI:** 10.3389/fped.2024.1351296

**Published:** 2024-09-10

**Authors:** Kai Hong, Jie Wan, Ying Zhao, Chao Zhang

**Affiliations:** ^1^ Department of Medical Ultrasound, Tongji Hospital of Tongji Medical College of Huazhong University of Science and Technology, Wuhan, China; ^2^ Institute of Pathology, Tongji Hospital of Tongji Medical College of Huazhong University of Science and Technology, Wuhan, China

**Keywords:** developmental dysplasia of the hip, ultrasound, acetabular, cartilage, hip

## Abstract

**Introduction:**

It has been reported that the cartilaginous roof of the acetabulum is thicker in infants with developmental dysplasia of the hip (DDH) than in those with healthy hips. However, there is limited research on the changes in the thickness of acetabular cartilage after follow-up or treatment of DDH. This study aims to report the thickness of acetabular cartilage before and after treatment of DDH.

**Materials and methods:**

In this prospective study, infants with clinical suspicion of DDH were enrolled in the pediatric outpatient service in our hospital from January 2022 to August 2023. The thickness of acetabular cartilage was measured in the standard coronal plane. Borderline hips (Graf IIa type) were monitored with monthly ultrasound examination until they were classified as normal hips (Graf I type), while dysplastic hips (Graf IIb type or worse) were treated with the Pavlik harness until they were also classified as normal hips in the final ultrasound examination.

**Results:**

A total of 592 children [median age, 96 days (interquartile range, 70–142 days); 197 boys] were enrolled in the study. The thickness of acetabular cartilage in dysplastic hips (4.3 ± 1.6 mm) was greater than that in normal hips (3.0 ± .39 mm, *P* < 0.001) and borderline hips (3.1 ± .57 mm, *P* < 0.001). In borderline hips, the thickness of acetabular cartilage decreased from 3.1 ± .57 mm in the initial evaluation to 2.9 ± .53 mm in the final follow-up scan (*P* = 0.01). In dysplastic hips, the thickness of acetabular cartilage decreased from 4.3 ± 1.6 mm in the initial evaluation to 3.5 ± .51 mm after treatment (*P* = 0.003). The thickness of acetabular cartilage in dysplastic hips after treatment remained greater than that in normal hips (*P* < 0.0001).

**Conclusion:**

The thickness of acetabular cartilage decreased after follow-up or treatment of DDH. Further research is required to determine whether cartilage that remain thicker in dysplastic hips than that in normal hips after treatment should be considered an early indicator of residual acetabular dysplasia.

## Introduction

Developmental dysplasia of the hip (DDH) includes a broad spectrum of disorders affecting the developing hip, ranging from subtle dysplasia detected by ultrasound and/or radiography without any clinical findings to a dislocated hip that can or cannot be reduced (1). DDH is a major cause of hip osteoarthritis, which can lead to severe disability in young adults (2). Clinical examination alone is not sensitive enough to identify every child with DDH. The sensitivity has been reported to be as low as 50% (1). Ultrasound has been widely used in screening DDH in infants younger than 6 months. The most commonly known ultrasound techniques used worldwide are the Graf (3) technique and the Harcke technique (4), which are recommended by clinical practice guidelines from several medical academies or institutions (1, 5, 6). With both ultrasound techniques, it was proven that hip sonography could detect abnormality not detected by clinical examination or radiograph (6, 7). However, universal screening by ultrasound may cause initial overtreatment without reducing the prevalence of surgical treatment (8, 9). According to the Graf method, treatment is required for any hip classified as type IIa minus or worse, i.e., with an alpha value of <60° by the end of the 12th week of life (3, 10). However, whether a hip with an alpha angle of slightly <60° should be treated is controversial (11). A study showed that many mild forms of DDH resolve without treatment (12). What's more, the interrater variability of Graf's alpha angle is problematically high (13), which may potentially alter the final diagnosis in 50%–75% of infants if scanned by a nonexpert (14).

If diagnosed and treated early, most cases of DDH are potentially reversible. Splints and braces, such as the Pavlik harness, to maintain abduction and flexing of the hips, are considered the gold standard for DDH treatment under 6 months of age with a reducible hip (15). Closed reduction and spica casts are the first-line treatment for a late-diagnosed dislocated hip (at >6 months of age). Open reduction is indicated when closed methods fail (16).

In infants with DDH, there is a hypertrophied ridge of acetabular articular cartilage and labrum in the superolateral aspect of the acetabulum (17, 18). Damage to the epiphyseal acetabular cartilage may hinder hip growth and development (18). Graf (3) considered that in a dysplastic hip, the wide as-yet unossified cartilage roof must finally ossify to become a normal joint. This makes acetabular cartilage thickness a potential indicator, in addition to the alpha angle, to describe pathological changes in DDH. Arthrographic indices (19), such as the cartilaginous acetabular index, enable assessment of the lateral labral margin. A cartilaginous acetabular index of >10◦ at the age of 5 years can predict poor acetabular development, but this modality is invasive (20). Ultrasound has been used to assess the cartilaginous roof of the acetabulum (21, 22). However, there is limited research on the changes in the thickness of acetabular cartilage after follow-up or treatment of DDH. This study aims to report the thickness of acetabular cartilage before and after treatment of DDH.

## Materials and methods

### Patients

**Figure 1:**
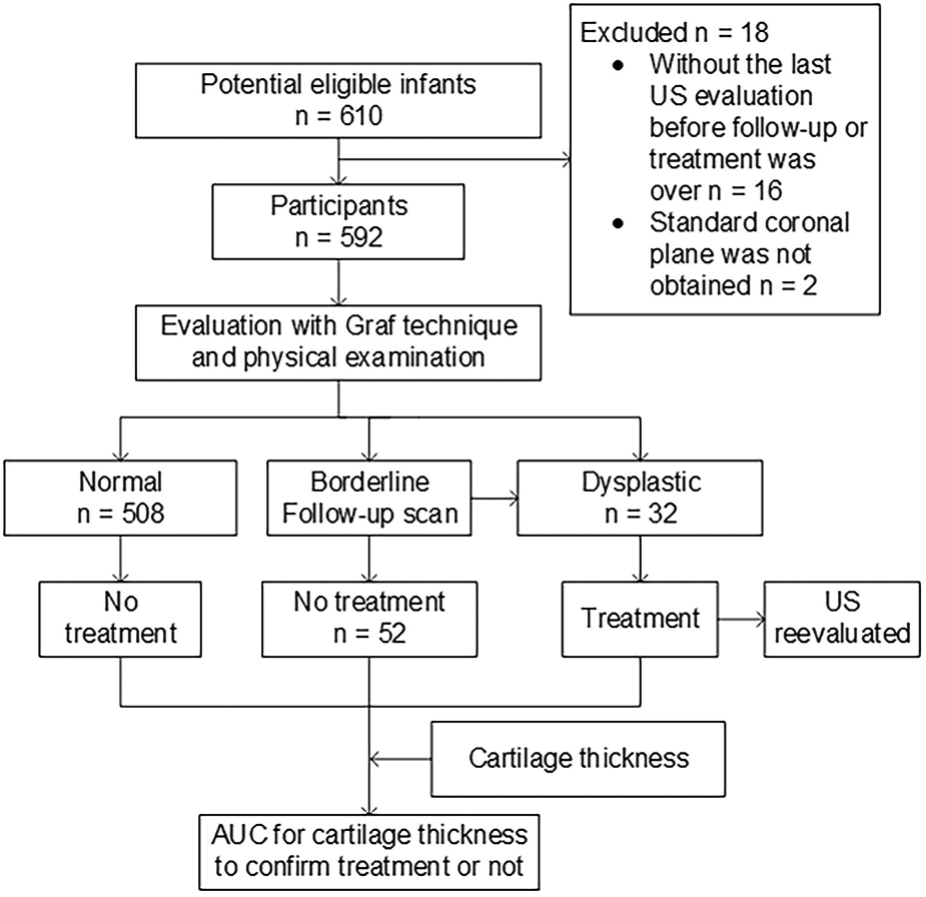
Flowchart of the study.

The study was approved by the institutional review board of our hospital and registered on the Chinese clinical trial registry website (http://www.chictr.org.cn, number ChiCTR2000040953). The datasets generated during and/or analyzed during the current study are available from the corresponding author upon reasonable request. The participants consisted of a consecutive sample of infants who met the predetermined inclusion criteria. Written informed consent was obtained from the parent(s) or guardian(s) of the infants. The infants were enrolled in the pediatric outpatient service in our hospital from January 2021 to August 2023. The inclusion criteria were infants with clinical suspicion of DDH, usually because of breech presentation, asymmetric folds, family history of developmental dysplasia, and a hip click. Borderline or dysplastic hips were followed up or treated by the Pavlik harness until they were classified as normal based on monthly ultrasound evaluation and physical examination. Infants were excluded if a standard coronal plane was not achieved in the ultrasound examination or if the last ultrasound evaluation of hips was not undertaken before the follow-up or treatment was over (Figure 1).

### Ultrasound imaging

Ultrasound of the hip was performed with an ultrasonic machine (Logiq E9, GE Medical Systems, or Philips EPIQ 7 system, Philips) equipped with a 7 or 10 MHz transducer.

Ultrasound examination of all infants was performed and interpreted by one radiologist (C.Z., with 10 years of experience in DDH evaluation). Part of the archived images of infants were reevaluated by another two radiologists (K.H. and Y.Z., with 5 and 2 years of experience in DDH evaluation, respectively) to test the interrater agreement of the measurement of the thickness of acetabular cartilage.

**Figure 2:**
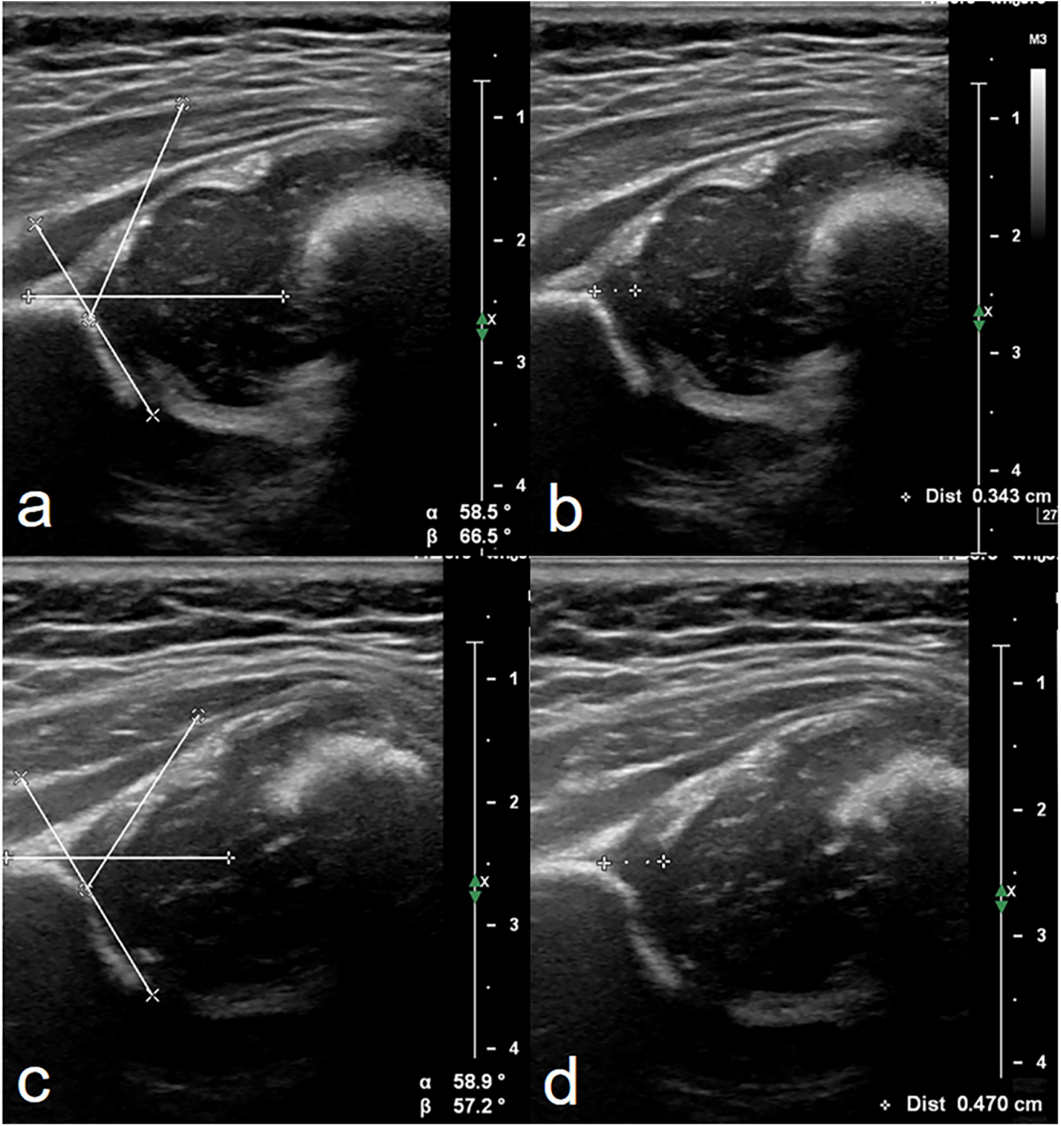
Measurement of alpha angle and the thickness of acetabular cartilage of hips in ultrasound images. Both were evaluated in the standard coronal plane described by Graf (10). Images **(a,b)** were from a 46-day-old female infant identified to be normal after a follow-up scan of hips 1 month later. Images **(c,d)** were from a 118-day-old female infant accepting treatment with the Pavlik harness for 1 month with a history of breech presentation. The same values of alpha angle (59°) were evaluated in these two patients. The thickness of acetabular cartilage in **d** was thicker than that in **b**. This provided more information to pediatric orthopedists in addition to alpha angle.

The ultrasound assessment of the hips was performed with the Graf technique (3, 9). The thickness of the acetabular cartilage was measured in the standard coronal plane. The intersection of the wing of the ilium and the bony roof of the acetabulum was considered the starting point for measurement. The thickness was measured along the baseline from the starting point to the boundary of the acetabular cartilage. Slight flexion and extension movement of the hip of the infant during ultrasound examination assisted by the investigator or guardian could make the boundary of the acetabular cartilage adjacent to the femoral head get clearer display (Figure 2, Supplementary Video 1).

### Thickness of acetabular cartilage

Relationships of the thickness of acetabular cartilage with age, length, and weight of infants were described. The thickness of acetabular cartilage in different types of hips was compared. Correlations between the thickness of acetabular cartilage and alpha angle were analyzed. Changes in the thickness of acetabular cartilage after follow-up scan in borderline hips or after treatment in dysplastic hips were described. Test performance characteristics by using the thickness of acetabular cartilage from the first scan to help detect DDH that required treatment were determined and compared with clinical reference standard diagnosis.

Interrater reliability was calculated (intraclass correlation coefficient) to confirm the reliability of the assessment of the thickness of acetabular cartilage.

### Statistical analysis

Statistical analyses were performed using SPSS 22.0 (IBM) and Prism 8.0 (GraphPad). The differences in the thickness of acetabular cartilage in different sex or age groups were tested with an independent-sample *t*-test, considering data were from the two independent groups. The differences in the thickness of acetabular cartilage from multiple groups, that is, the groups of normal hips, borderline hips, and dysplastic hips, were tested with ANOVA. Pearson correlation analysis was used for the assessment of the correlation between the thickness of acetabular cartilage and length, weight, or alpha angle. The changes in cartilage thickness before and after follow-up scan or treatment were analyzed by a paired-sample *t*-test, considering the data were from the same group of samples. *P* < 0.05 was considered to indicate a statistically significant difference.

## Results

### Patient characteristics

A total of 592 infants [median age, 96 days (interquartile range, 70–142 days); 197 boys] were enrolled in the study. The cohort included those with normal hips (*n* = 508), borderline hips (Graf IIa, later normalizing spontaneously; *n* = 52), or dysplastic hips (Graf IIb or worse, *n* = 32) clinically diagnosed after more than 6 months of follow-up (Figure 1).

### Relationships of cartilage thickness with sex, age, length, and weight in infants with normal hips

There was no difference in the thickness of acetabular cartilage between the boys (2.9 ± .39 mm, *n* = 114) and the girls (3.0 ± .38 mm, *n* = 394) (*P* = 0.31). Infants with normal hips were divided into two groups according to age less than or more than 90 days, which was considered the age for hips to reach maturity (1, 10). There was no difference between the thickness of acetabular cartilage in younger infants (3.1 ± .41 mm, *n* = 110) and that in elder infants (3.0 ± .39 mm, *n* = 398) (*P* = 0.12). No correlation between the thickness of acetabular cartilage with length (Pearson *r* = 0.11, *P* =0 .21) or weight (Pearson *r* = .12, *P* = 0.16) of infants was found.

### Thickness of acetabular cartilage in different types of hips

**Figure 3:**
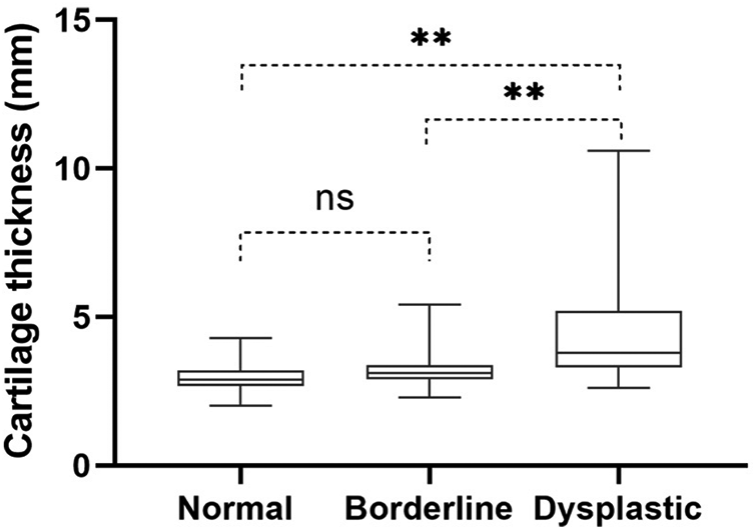
The thickness of acetabular cartilage in normal hips, borderline hips, and dysplastic hips. ns, no significant differences, **, *P* < 0.001.

There was no difference in the thickness of acetabular cartilage between normal hips (3.0 ± .39 mm) and borderline hips (3.1 ± .57 mm, *P* = 0.09). The thickness of acetabular cartilage in dysplastic hips (4.3 ± 1.6 mm) was greater than that in normal hips (*P* < 0.001) and borderline hips (*P* < 0.001) (Figure 3).

### Correlation between the thickness of acetabular cartilage and alpha angle

**Figure 4:**
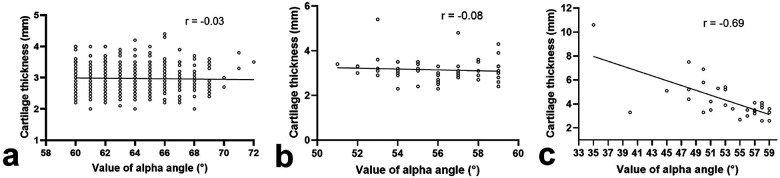
Correlation between the thickness of acetabular cartilage and alpha angle in normal hips **(a)**, borderline hips **(b)**, or dysplastic hips **(c)**.

No correlation between the thickness of acetabular cartilage and alpha angle was found in normal hips (*P* = 0.50, Figure 4a) or borderline hips (*P* = 0.58, Figure 4b). In dysplastic hips, the thickness of acetabular cartilage increased with the decrease in alpha angle (*P* < 0.001, Figure 4c).

### Thickness of acetabular cartilage before and after follow-up or treatment

**Figure 5:**
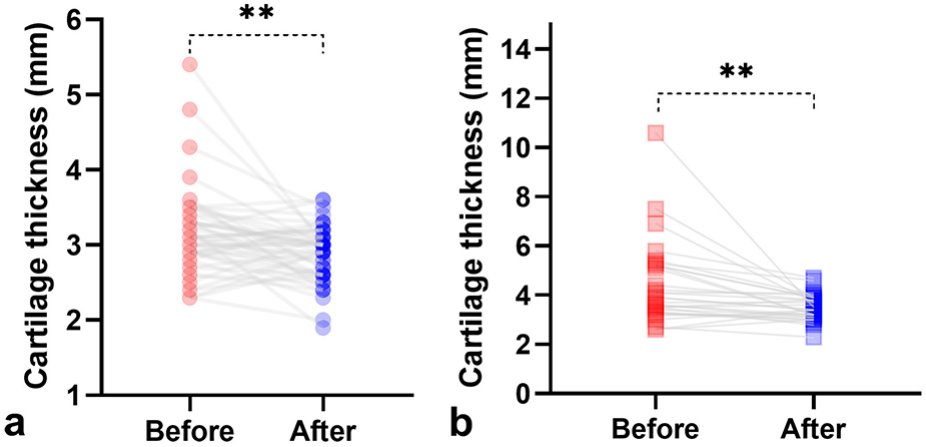
Comparison of the thickness of acetabular cartilage between the primary and the last ultrasound evaluation. The thickness of acetabular cartilage deceased after follow-up in borderline hips **(A)** or after treatment in dysplastic hips **(B)**. **P < 0.01.

The thickness of acetabular cartilage evaluated in the last ultrasound examination was compared with that in the first examination for hips that were followed up or treated. In borderline hips, the thickness of acetabular cartilage decreased from 3.1 ± .57 mm in the first evaluation to 2.9 ± .53 mm in the last follow-up scan (*P* = 0.01). In dysplastic hips, the thickness of acetabular cartilage decreased from 4.3 ± 1.6 mm in the initial evaluation to 3.5 ± .51 mm after treatment (*P* = 0.003) (Figure 5). The thickness of acetabular cartilage in dysplastic hips after treatment remained greater than that in normal hips (*P* < 0.0001).

### AUC of cartilage thickness to determine treatment or not

**Figure 6:**
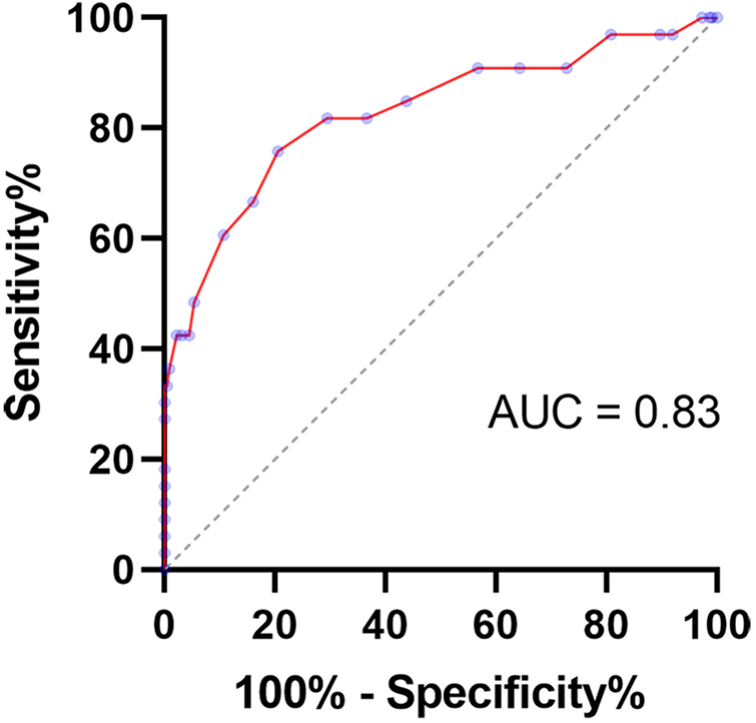
Receiver operating characteristic curve for the thickness of acetabular cartilage to detect hips that required treatment.

Receiver operating characteristic curves were generated (Figure 6) for the thickness of acetabular cartilage from the first scan to help detect dysplastic hips that required treatment. The area under the receiver operating characteristic curve was 0.85 (95% CI, 0.77–0.93, *P* < 0.0001). With a cutoff value of 3.3 mm (the value with the highest Youden index), the thickness of acetabular cartilage can detect dysplastic hips with a sensitivity of 81.3% and a specificity of 73.9%.

### Interrater reliability

Ultrasound images from 50 hips were randomly selected for reevaluation of the thickness of acetabular cartilage. The interrater repeatability of the intraclass correlation coefficient was 0.91 [(95% CI: 0.86–0.95), *P* < 0.001].

## Discussion

The thickness of the acetabular cartilage of infants was evaluated in this study. We confirmed that the thickness of acetabular cartilage in dysplastic hips was greater than that in normal hips. The thickness of acetabular cartilage in borderline hips and dysplastic hips decreased after follow-up or treatment.

The mean thickness of acetabular cartilage in normal hips we measured (3.0 mm) is greater than that of the cartilage measured by Soboleski and Babyn (2.6 mm) (21). This is due to the different measuring methods used in the two studies. Soboleski and Babyn used the apex of the alpha angle as the starting point for measuring the thickness of cartilage (21). This may underestimate the thickness of acetabular cartilage in a considerable part of immature hips (e.g., Figure 2c).

In our study, we confirmed that the more severe the dysplasia, the greater the thickness of acetabular cartilage. Tréguier et al. (22) reported 15 irreducible neonatal dislocated hips. The thickness of acetabular cartilage was between 5 and 7 mm, which was greater than the mean thickness (4.3 mm) of dysplastic hips in our study. This is because 97% (31/32) of dysplastic hips in our study are non-dislocated hips, with relatively mild dysplasia compared to dislocated hips. In dysplastic hips, articular hyaline cartilage may also contribute to the thicker cartilage, which was confirmed by Nishii et al. (23) using MRI in patients aged 16–44 years, considering the physis and epiphysis in the adult patients should have already been ossified.

The thickness of acetabular cartilage decreased after follow-up in borderline hips or treatment in dysplastic hips. This change in cartilage thickness reflects the gradual ossification of the cartilage over time, which is promoted by harness treatment in a direction conducive to the normalization of acetabulum morphology. The cartilage thickness in dysplastic hips after treatment (3.5 ± .51 mm) was still greater than that in normal hips (3.0 ± .42 mm). Some cartilaginous indicators based on MRI or hip arthrography have been proposed to be used as early warning indicators of residual acetabular dysplasia (24). The residual acetabular dysplasia may require acetabuloplasty to protect the hip in the later stage (24) or total hip arthroplasty in adulthood (25). Whether the still thicker cartilage measured by ultrasound should be considered as an indicator of residual acetabular dysplasia needs to be further studied.

There are some issues that need to be addressed. First, the acquisition of ultrasound images was performed by a single operator, and the interexaminer reliability still needs to be clarified. In future studies, acetabular cartilage should be imaged by multiple ultrasound operators to determine the consistency between different examiners. Second, the thickness of acetabular cartilage in our study was measured in the superior lateral part of the cartilaginous roof of the acetabulum, which was evaluated in the standard coronal plane. However, in some dislocated hips, the standard coronal plane may not be achievable by ultrasound. In such cases, the thickness of the acetabular cartilage could be measured after the hips are reduced.

## Conclusions

The thickness of acetabular cartilage decreased after follow-up or treatment of DDH. Further research is required to determine whether cartilage that remain thicker in dysplastic hips than that in normal hips after treatment should be considered an early indicator of residual acetabular dysplasia.

## Data Availability

The raw data supporting the conclusions of this article will be made available by the authors, without undue reservation.

## Supplementary material

Supplementary Video 1 Slight movement of the infant's thigh during ultrasound imaging of the hip may facilitate recognition of the boundary of acetabular cartilage.
